# Initial Steps in Mammalian Autophagosome Biogenesis

**DOI:** 10.3389/fcell.2018.00146

**Published:** 2018-10-23

**Authors:** Daniel Grasso, Felipe Javier Renna, Maria Ines Vaccaro

**Affiliations:** Institute of Biochemistry and Molecular Medicine (IBIMOL-CONICET), School of Pharmacy and Biochemistry, University of Buenos Aires, Buenos Aires, Argentina

**Keywords:** autophagy regulation, mTOR, AMPK, ULK1, VMP1

## Abstract

During the last decade, autophagy has been pointed out as a central process in cellular homeostasis with the consequent implication in most cellular settings and human diseases pathology. At present, there is significant data available about molecular mechanisms that regulate autophagy. Nevertheless, autophagy pathway itself and its importance in different cellular aspects are still not completely clear. In this article, we are focused in four main aspects: (a) *Induction of Autophagy*: Autophagy is an evolutionarily conserved mechanism induced by nutrient starvation or lack of growth factors. In higher eukaryotes, autophagy is a cell response to stress which starts as a consequence of organelle damage, such as oxidative species and other stress conditions. (b) *Initiation of Autophagy*; The two major actors in this signaling process are mTOR and AMPK. These multitasking protein complexes are capable to summarize the whole environmental, nutritional, and energetic status of the cell and promote the autophagy induction by means of the ULK1-Complex, that is the first member in the autophagy initiation. (c) *ULK1-Complex:* This is a highly regulated complex responsible for the initiation of autophagosome formation. We review the post-transductional modifications of this complex, considering the targets of ULK1. (d)*The mechanisms involved in autophagosome formation*. In this section we discuss the main events that lead to the initial structures in autophagy. The BECN1-Complex with PI3K activity and the proper recognition of PI3P are one of these. Also, the transmembrane proteins, such as VMP1 and ATG9, are critically involved. The membrane origin and the cellular localization of autophagosome biogenesis will be also considered. Hence, in this article we present an overview of the current knowledge of the molecular mechanisms involved in the initial steps of mammalian cell autophagosome biogenesis.

There are three types of autophagy, processes where cytoplasmic components are delivered to lysosomes for degradation: microautophagy/endosomal microautophagy (Li et al., 2012; Galluzzi et al., 2017), chaperone-mediated autophagy (CMA) (Cuervo and Wong, 2014; Kaushik and Cuervo, 2018) and macroautophagy (hereafter mentioned as autophagy). This is the engulfment of cytoplasmic contents by a double membrane vesicle, named autophagosome. The outer membrane of the autophagosome eventually fuses with the lysosome, where the inner vesicle is delivered (Figure 1). Here we present a brief overview of the mechanisms involved in the initial steps of mammalian cell autophagosome biogenesis.

**FIGURE 1:**
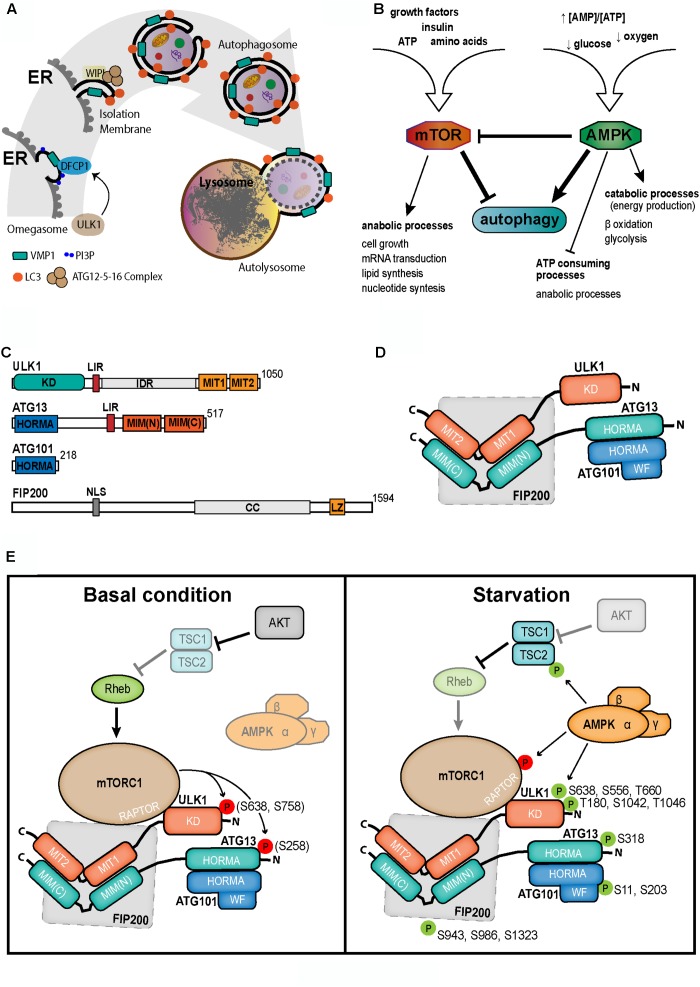
**(A)** Schematic overview of autophagy. UKL1 activation leads to autophagosome biogenesis. On the ER surface, the transmembrane protein VMP1 recruits a PI3K complex. The consequent PI3P subdomain is recognized by DFCP1 on the omegasome structure. Then, in the isolation membrane, WIPI proteins recruit the ATG5-ATG12-ATG16 complex which in turn make possible the lipidation of LC3 on the membrane. The formation of autophagosome, a double membrane vesicle, allows the carrying of cargo to lysosome. Eventually, cargo is degraded in the resulted autophagolysosome. ER, endoplasmic reticulum; PI3K, phosphatidylinositol 3-kinase; PI3P, phosphatidylinositol (3,4,5) triphosphate (PI3P). **(B)** Diagram of interrelationship among the cellular energetic and metabolic regulators, mTOR and AMPK, and the autophagy. **(C)** Representative scheme of the ULK1 complex proteins. Upper right number in each scheme shows the length of the amino acid chain. Described domains are showed for each protein. **(D)** Possible structure and interrelationship among the ULK1 complex proteins, suggested from available data. KD, kinase domain; LIR, LC3-interacting region; IDR, intrinsically disordered region; MIT, microtubule interacting and trafficking domain; HORMA, HOP1, REV7, and MAD2 domain; MIM, MIT-interacting motif; NLS, nuclear localization signal; CC, coil-coil region; LZ, leucine zipper; WF, WF finger motif. **(E)** Regulation of the autophagy initiation complex ULK1 by mTOR and AMPK at basal (left) and starvation (right) conditions.

## Induction of Autophagy

The main task of autophagy is to deal against poor nutrient environments. In superior eukaryote cells, mTOR, which is a serine/threonine kinase, checks the presence of growth factors and nutrients. In presence of amino acids (mainly leucine, glutamine and arginine), mTORC1 maintains the autophagy inhibition. When nutrients are no longer available, the inhibition of mTORC1 releases the ‘brake’ and autophagy is eventually induced (Carroll et al., 2016). Growth factors negatively regulate the autophagy by activation of mTOR. Activation of the insulin receptor induces the phosphorylation of TSC2, avoiding the TSC1/2 complex formation and the mTORC1 inhibition (Haeusler et al., 2018). Other growth factors induce the RAS pathway, which activates the ERK1/2 dimer that inhibits the TSC1/2 complex and phosphorylates RAPTOR activating mTORC1 and suppressing autophagy (Carriere et al., 2011).

AMPK is a key serine/threonine kinase that is activated in low energy conditions (Egan et al., 2011). Then, AMPK activates the autophagosome formation by mean of direct and indirect ways. Furthermore, AMPK can be activated by CaMKKB in the ER-overloaded response (Hoyer-Hansen et al., 2007). The unfolded protein response, by mean of IRE1α, PERK and ATF6, is also an autophagy triggering event, enhancing LC3 conjugation (Ding et al., 2007; Kouroku et al., 2007).

During quick and intense oxygen fluctuations, autophagy is induced by mTORC1-dependent pathways and/or by ER stress. (Papandreou et al., 2008; Rouschop et al., 2010). In moderate but chronic hypoxia, autophagy is triggered mainly by HIF1α and PKCδ-JNK1 pathways (Mazure and Pouyssegur, 2010). HIF1α is the major transcription factor involved in cell response to hypoxia (Brocato et al., 2014). Among the genes transcribed by HIF1α is BNIP3 which disrupts the Bcl2-BECN1 interaction releasing BECN1 to be part of the autophagy process (Zhang et al., 2008), and VMP1, which interacts with BECN1 and is required for autophagosome formation (Ropolo et al., 2007; Rodriguez et al., 2017). Regarding to the PKCδ pathway, this kinase activates JNK1 that in turn phosphorylates Bcl2 to release it from BECN1 (Pattingre et al., 2009).

Oxidative stress induces autophagy in order to recycle damaged mitochondria (and other damaged organelles), and eliminate proteins aggregates (Ureshino et al., 2014). NRF2 is bound to antioxidant response elements promoting the transcription of p62, a cargo receptor for autophagy (Puissant et al., 2012). FOXO3 induces the expression of LC3 (an ATG protein that is described below) and BNIP3 (Mahalingaiah and Singh, 2014). Finally, ROS inhibit ATG4-mediated LC3 delipidation, that takes place immediately after formation of the autolysosome, conferring stability to LC3 and favoring its recruitment to the autophagosome (Scherz-Shouval et al., 2007).

## Initiation of Autophagy

Independently of the induction agent, in canonical autophagy, the initiation of autophagosome biogenesis is managed by the kinases mTOR and AMPK. In fact, through the association with RAPTOR, DEPTOR, PRAS40 and mLST8, mTOR constitutes the complex 1 [mTORC1]. At basal conditions, mTORC1 is stimulated by the small GTPase Rheb. In turn, mTOR triggers cell growth and diverse anabolic processes such as lipids, proteins and nucleotides synthesis (Lamb et al., 2013; Klionsky and Schulman, 2014). On the other hand, active mTORC1 abolishes most of catabolic processes including the autophagy (Lamb et al., 2013; Klionsky and Schulman, 2014; Figure 1B). Therefore, mTOR inhibits autophagy, by several phosphorylations on the first complex of the pathway (see further), when optimal nutrients concentration is available.

During starvation, Rheb is inhibited by the TSC1/2 heterodimer removing the activation stimulus on mTOR (Huang and Manning, 2008). This inhibition of mTORC1 decreases its influence on autophagy and as a consequence, the mechanism of autophagosome biogenesis is triggered (Carroll et al., 2016; Figure 1E). Moreover, the inactivation of mTORC1 allows that the dephosphorylated TFEB translocates to the nucleus (Puertollano et al., 2018) where it induces the transcription of ATG genes, such as UVRAG, WIPI, MAPLC3B, SQSTM1, Vps11, Vps18, and ATG9B. TFEB also promotes the lysosomal function in the cell (Settembre et al., 2011).

AMPK is a heterotrimeric complex composed by a catalytic α subunit and two regulatory subunits, β and γ (Egan et al., 2011). Since AMPK is activated in low energy conditions, this kinase inhibits anabolic processes, and induces catabolic pathways, such as autophagy (Egan et al., 2011; Zhang et al., 2013; Figure 1B). AMP binding allows LKB1 to phosphorylate AMPK (Thr172) (Xiao et al., 2007; Zhang et al., 2013), which in turn directly and indirectly activates the autophagosome formation as is explained in the next sections.

## Ulk1 Complex

ULK1 is so far the first complex in the core molecular machinery involved in the biogenesis of autophagosomes. This complex is composed by the serin/threonin protein kinase ULK1, ATG13, FIP200, and ATG101. Activated ULK1 is capable of triggering series of phosphorylations that enable the nucleation process and autophagosome biogenesis. At N-terminal ULK1 is the kinase domain followed by a disordered region that is postulated as highly regulated. On the opposite side, there are two MIT domains in tandem that compose a globular structure (Noda and Fujioka, 2015). ULK1 structure was characterized in complex with ATG13. On the C-terminal of ATG13 there are two MIT-interacting motifs in a helical region for recognition-interaction with the ULK1 MIT domains (Noda and Fujioka, 2015; Qi et al., 2015). Additionally, both proteins, ULK1 and ATG13, have a LIR domain for interaction with LC3 family members. ATG101, the smallest member of the complex, is essential for autophagy (Mercer et al., 2009). ATG101 is almost fully composed by a HORMA domain with direct interaction with the HORMA domain at the N-terminus of ATG13. ATG101 stabilizes ATG13 and ULK1 (Mercer et al., 2009; Suzuki et al., 2015) and seems to recruit downstream molecules through its WF finger motif (Suzuki et al., 2015). The last member of ULK1-complex is FIP200, that is the largest molecule involved in this complex (Hara et al., 2008; Figures 1C,D).

ULK1 complex is regulated by the two major key proteins related to nutritional and energetic sensing, mTOR and AMPK (He and Klionsky, 2009). Under growth factors stimulation and nutrient availability, the activated mTORC1 interacts with ULK1 through RAPTOR and phosphorylates several sites of ULK1 (Ser757/5637 in mouse, Ser758 in human) (Alers et al., 2012) and Atg13 (Ser258 in mouse) subunits (Kim et al., 2011; Puente et al., 2016). Then, ULK1 complex remains inactivated and autophagy repressed. AMPK induces ULK1-mediated autophagy by three strategies: 1- AMPK phosphorylates TSC2 at Ser1345 enhancing the activity of this mTORC1 inhibitor (Inoki et al., 2003). 2- AMPK is able to inhibit mTORC1 activity directly by phosphorylation of Raptor in Ser792/722 (Gwinn et al., 2008; Egan et al., 2011). 3- AMPK interacts with and phosphorylates ULK1 in Ser317/777 for its activation (Kim et al., 2011; Figure 1E).

Another pathway for ULK1 autophagy activation has been proposed: AMBRA1 may act as a bridge between ULK1 and the ubiquitin ligase E3 TRAF6 (Nazio et al., 2013; Grumati and Dikic, 2018). TRAF6-mediated poly ubiquitination, K63 type branched ubiquitin, potentiates autophagy activation by promoting stabilization and self-association of ULK1. This event initiates a positive loop, where ULK1 phosphorylates AMBRA1 enhancing TRAF6-mediated ULK1 ubiquitination (Nazio et al., 2013; Grumati and Dikic, 2018). Further, growth factors withdrawal might induce the activation of TIP60 by GSK3-mediated phosphorylation at Ser86. TIP60 is an acetyltransferase that induces the activation of ULK1 by acetylation of Lys162/606 enhancing the triggering of autophagy (Lin et al., 2012).

## The Mechanisms Involved in Autophagosome Biogenesis

Once activated, ULK1 is able to phosphorylate several substrates. Among them, there are two initial complexes, the ULK1 complex itself and the PI3KC3 complex 1 (PI3KC3-C1). In the first complex, ULK1 phosphorylates to itself (Thr180/1046, Ser1042) (Bach et al., 2011), and the other members of the complex, Atg13 (Ser318/203), FIP200 (Ser943/986/1323) and ATG101 (Ser11/203) (Lin and Hurley, 2016; Orhon and Reggiori, 2017; Figure 1E). In the second complex, ULK1 potentiates the PI3K activity of the catalytic subunit Vps34, by the phosphorylation of two members of the complex, BECN1 (Ser14) and ATG14L (Ser29), resulting in the increment of PI3P production (Russell et al., 2013). Following to ULK1 complex activation, the transmembrane protein VMP1 interacts with the BH3 domain of BECN1 through its ATG domain, recruiting the PI3KC3-C1 to the autophagosomal membrane (Molejon et al., 2013).

There are two main PI3KC3 complexes in autophagosome biogenesis. The complex 1 is composed by BECN1, ATG14L, Vps15 and Vps34, which is a key component in autophagosome initiation. The other complex, PI3KC3-C2, is related to autophagosome maturation and endosomal trafficking and is composed by the same members except for the regulatory protein ATG14L which is replaced by UVRAG. Structurally, the PI3KC3-C1 is stabilized in pairs, BECN1/ATG14L and Vps15/Vps34 (Stjepanovic et al., 2017). Upon autophagy induction, BECN1 recruitment induces the complex assembly, through the adaptor ATG14L, where the WD domain of Vps15 organizes the proteins into the complex allowing the activity of Vps34 (Stjepanovic et al., 2017). Moreover, the KAP1-mediated SUMOylation of Vps34 enhances the interaction of this protein with the rest of the complex (Yang et al., 2013). As it was commented before, ULK1-mediated phosphorylation of BECN1, ATG14L and Vps34 potentiates PI3K activity in this complex. The tumor suppressor DAPK, a calcium/calmodulin serine/threonine kinase, also contributes to the PI3KC3-C1 recruitment to the autophagosome membrane. This kinase phosphorylates BECN1 on its BH3 domain interfering with the BECN1-Bcl-xL association and releasing BECN1 (Zalckvar et al., 2009). This effect is reaffirmed by TRAF6 which ubiquitinates BECN1 on the same region (Shi and Kehrl, 2010). Recently, it has been proposed that Vps34 activity may be switched on/off by an EP300-dependent acetylation/deacetylation on K771, as another regulation of the PI3KC3-C1 (Su and Liu, 2017; Su et al., 2017).

The cascade of subsequent activations of ULK1 and PI3KC3-C1 complex members is limited by a series of degradative processes. The deubiquitinase A20 (DUB A20) controls BECN1 participation on autophagosome formation by elimination of poly ubiquitin chain in the BH3 domain placed by ATF6 E3 ligase. Beyond that regulation, the E3 ligases NEDD4 and NEDD4L induce degradation of key members in ULK1, and Vps34 complexes respectively (Platta et al., 2012; Nazio et al., 2016). BECN1 is poly ubiquitinated with K11-linked ubiquitin chain by NEDD4 to be eliminated in the proteasome. Similar activity is carried out by NEDD4L on ULK1 targeting this protein with K27- and K29-linked ubiquitin chains. In both cases, the proteasome-mediated elimination of those proteins causes the destabilization of its respective complexes. In a redundant way of labeling for degradation, the poly ubiquitination with K48-linked ubiquitin chains on ULK1, BECN1, and Vps34 is catalyzed by the complex CUL3-KHLH20 (Liu and Chen, 2016).

### The Omegasome and the Isolation Membrane

The local enrichment of PI3P in ER-subdomains acts as the signal for the nucleation of several autophagy-related proteins in a structure named omegasome that resembles the Greek letter omega (Ktistakis and Tooze, 2016). The first protein which recognizes the PI3P is DFCP1. DFCP1 possesses a diffuse pattern over the ER, mitochondria and Golgi but it is rapidly mobilized to the PI3P spots by the recognition of this phospholipid with the two FYVE motifs of its structure. Although it is a marker of omegasome, little is known about its role during the initial steps of autophagosome biogenesis. Additionally, the DFCP1 depletion does not seem to interfere with the progression of autophagy.

The rising omegasome leads to extension of a sack-like structure named isolation membrane or phagophore. WIPI2b, a member of the PROPPIN family, recognizes the local PI3P by the FRRG motif of its WD40-repeat β-propeller on the isolation membrane (Nascimbeni et al., 2017a). The process continues with two ubiquitin like systems: ATG12 and LC3. Cytoplasmic ATG12 is covalently attached to a C-terminal glycine of ATG5. This catalytic reaction resembles the ubiquitination process where ATG7 and ATG10 are subrogated to E1 and E2 enzymes, respectively (Klionsky and Schulman, 2014). ATG5-ATG12 complex is highly important, since it functions as E3 enzyme for LC3 conjugation to phosphatidylethanolamine (PE) on the autophagosomal membrane. This process seems to be mediated by ATG16L, which is composed by a WD40-repeat β-propeller domain localized in the C-terminal sequence. At N-terminal sequences, ATG16L possesses a binding domain that allows the interaction with ATG5 to eventually form the ATG12-ATG5-ATG16L complex (Wilson et al., 2014). The middle sequence of ATG16L expands a coil-coil (cc) dimerization domain that induces the formation of ATG16L dimers (Wilson et al., 2014). Then, WIPI2b is recognized by a region of ATG16L, between the cc-dimerization domain and the WD40-repeated β-propeller domain. Consequently, the ATG12-ATG5-ATG16L complex is recruited to the isolation membrane. LC3 plays a central role in autophagy being involved in vesicle elongation, maturation, fusion of autophagosome-lysosome and even as an adaptor to cargo recognition (Nakatogawa et al., 2007; Lee and Lee, 2016). LC3 shows a diffuse pattern distributed over the cytoplasm and into the nucleus (known as LC3-I) in basal conditions. Upon autophagy triggering, LC3 is deacetylated in the nucleus by SIRT1 (Huang et al., 2015) and is cleaved in cytoplasm by ATG4B, which eliminates the C-terminal arginine residue to expose a glycine (Satoo et al., 2009; Maruyama and Noda, 2017). In an ubiquitin-like reaction, the exposed glycine is combined to form a thioester bound, first with ATG7 (E1-like enzyme) and then with ATG3 (the E2-like enzyme) (Satoo et al., 2009; Maruyama and Noda, 2017). ATG3 is recognized by ATG12 of the ATG12-ATG5-ATG16L complex which has been already recruited to isolation membrane through WIPI2b. The ATG12-ATG5-ATG16L complex functions as the E3 enzyme leading the formation of an amide bound with the amine headgroup of PE (Noda et al., 2013; Otomo et al., 2013; Dooley et al., 2015). The lipidated LC3 (LC3-II) is present at the isolation membrane and on the autophagosome, in both sides of the membrane. The arrival of autophagosome to the lysosome is a fusion dependent mechanism of the HOPS complex, through STX17 (Jiang et al., 2014), and RAB7 (Gutierrez et al., 2004). Since LC3 is present in both membranes of autophagosome, once exposed to lysosomal hydrolases, there is a pool of LC3 that is degraded with cargo. However, the LC3 localized in the external membrane is cleaved from the PE, by ATG4B, and then recycled. (Noda et al., 2013; Otomo et al., 2013; Dooley et al., 2015).

### Autophagosome Biogenesis in Non-Canonical Autophagy

Furthermore, of which is explained above, autophagy is able to follow unconventional pathways. ER-stress or glucose influx after starvation in NIH3T3, can induce autophagy independent of mTOR inhibition and where AMPK activation is not essential (Corona Velazquez and Jackson, 2018). Moreover, the glucose influx in mouse embryonic fibroblast can trigger autophagy independent of ULK1/2. Starved chicken DT40 cells show an autophagy dependent of ATG13-FIP200 interaction but independent of ULK1. Similar behavior is observed in some viral infection, such as coronaviruses, HBV or Poliovirus, which induce a non-degradative ULK1-independent form of autophagy. Even more interesting is that the oleate fatty acid can induce an autophagy mechanism that lacks of PI3P synthesis, since it cannot be inhibited by knocking-down of BECN1, Vps34, or ATG14. These examples suggest that autophagy is flexible and the pathways in autophagosome biogenesis may adapt to different situations depending on the inductor and the biological context (Corona Velazquez and Jackson, 2018).

### Autophagosome Initiation Site

It is accepted that the initial structure related to autophagy is located on the ER. The data suggest that ULK1 complex translocates to phosphatidylinositol-enriched ER-subdomains and then, the membrane structure is fed by ATG9A-containing vesicles (Nishimura et al., 2017). Then, autophagosomes are formed in highly active ER-subdomains where lipidic interchange between ER and other cytoplasmic organelles occurs.

Two sites of autophagosome biogenesis have been recently demonstrated: The ER-plasma membrane contact site (ER-PM) and the ER-Mitochondria contact site (Hamasaki et al., 2013; Nascimbeni et al., 2017b). VMP1 is a key player in the biogenesis of autophagosomes that remains in the autophagosomal membrane (Grasso et al., 2011). VMP1-BECN1 interaction allows the recruitment of PI3KC3-C1 to the ER-PM contact site by the interaction with the proteins Esyt 1, 2, and 3 (Nascimbeni et al., 2017b). Moreover, VMP1 was suggested to also regulate the ER-mitochondria contact site during autophagy and to be involved in the release of the initial autophagosome vesicle by activation of SERCA pump (Tabara and Escalante, 2016; Zhao et al., 2017). The transmembrane protein ATG9A is in Golgi and endosomal system, in early and late endosomes with a minimal percentage of recycling ones (Feng and Klionsky, 2017). In starvation, the TRAPPIII complex, related to ER-Golgi vesicular trafficking, mobilizes ATG9A vesicles to the sites of nascent autophagosomes (Shirahama-Noda et al., 2013). The adaptor protein AP-4 is required for this event, since it mediates the trafficking of ATG9A from *trans-*Golgi network to the site of autophagosomes maturation (Mattera et al., 2017). This event would potentiate the expansion of the isolation membrane. Nevertheless, the contribution of this membrane by the ATG9A vesicles is not enough to explain the growth of the membrane itself. Moreover, ATG9 seems to take a distinctive role in different systems. In contrast to mammals, yeast ATG9 has a fundamental role at very early steps in the pre-autophagosomal structure. On the other hand, in plants, the depletions of *Arabidopsis* ATG9 still allows formation of autophagosomal structures supplemented with ATG8 (LC3 ortholog) suggesting divergent regulation and mechanisms of this types of vesicles (Zhuang et al., 2017).

Ribosomes-free regions specialized in ER-Golgi communication are present in the rough ER. Vesicles arise targeted to the Golgi from these areas, described as ER-exit sites (ERES). These vesicles are supplemented by the proteins Sar1, Sec23, Sec24, Sec13 and Sec31, that constitute the COPII coat (Zahoor and Farhan, 2018). Before reaching Golgi, the COPII-coated vesicles go through an intermediated structure named ER-Golgi intermediate compartment (ERGIC) (Ben-Tekaya et al., 2005). The function of these structures is not completely understood, but they might participate in the autophagosome biogenesis. An impairment of these compartments causes an autophagy downregulation (Karanasios et al., 2016; Zahoor and Farhan, 2018).

Data suggest that the bulk contribution for the growth of the autophagosome membrane comes from the ER-Golgi vesicular trafficking. During starvation, the FIP200-CTAGES5 interaction induces the remodeling and enlargement of ERES positives for Sec12 (Ge et al., 2017). This allows the production of COPII-coated vesicles that are released to contribute to autophagosome formation. Moreover, ULK1 phosphorylates Sec23A, a member of the COPII multiprotein complex. This event is related to morphological variations on ERES during starvation and might turn the secretory machinery from anabolic to catabolic state.

A recent work shows a previously unexpected key role of Rab11A-positive membranes in autophagosome biogenesis (Puri et al., 2018). They demonstrated that WIPI2 relies, beyond the recognition of PI3P, in the interaction with Rab11A for recruitment of ATG16L. Also, the authors suggest a model where isolation membrane is represented by Rab11A-positive membrane, likely to be recycling endosomes. In this context, Rab11A-positive membranes constitute the platform for autophagosome formation initial steps.

## Conclusion and Perspectives

The initial molecular steps in autophagosome biogenesis are determined by three mains complexes: ULK1 complex; PI3KC3-C1; and ATG16L1–ATG5–ATG12 which eventually favors LC3 lipidation in the growing isolation membrane. LC3 family seems to play a relevant role in cargo recognition, autophagosome closure and fusion with lysosomes. However, while the initial molecular steps seem to be essential and well-known in canonical autophagy, the subsequent events in mammalian autophagosome biogenesis are less characterized. Moreover, the wide spectrum of autophagy-related events and the number of molecules involved (Table 1) leads to the concept that different pathways might account for diverse types of autophagy and may reveal different functions of autophagy in physiological and pathological cellular processes. Furthermore, the meaning of different origins and composition of the autophagosomal membrane, such as those supplied by ATG9A and COP-II vesicles (Feng and Klionsky, 2017), are still not fully understood.

**Table 1:** Main molecules involved in the initial steps of mammalian autophagosome biogenesis.

Protein	Complete name	Autophagy related function	Reference
mTOR	Mammalian target of rapamycin	Members of mTOR complex 1 (mTORC1): Autophagy inhibition by phosphorylation of ULK1 complex	Lamb et al., 2013
RAPTOR	Regulatory-associated protein of mTOR		
DEPTOR	DEP domain containing mTOR-interacting protein		
PRAS40	Proline-rich AKT1 substrate 40		
mLST8	Mammalian lethal with SEC13 protein 8		
AMPK	AMP-activated protein kinase	Autophagy activation by ULK1, mTORC1, and TSC2 phosphorylation	Egan et al., 2011
p62	Sequestosome-1 (SQSTM1 gene)	Autophagy cargo receptor	Puissant et al., 2012
ULK1	Unc-51-like kinase 1	Members of ULK1 complex	Bach et al., 2011; Russell et al., 2013
ATG13	Autophagy-related protein 13		
FIP200	FAK family interacting protein of 200 kDa		
ATG101	Autophagy-related protein 101		
BECN1	Beclin 1	Members of PI3KC3-C1/2	Ktistakis and Tooze, 2016
Vps15	Serine/threonine-protein kinase VPS15		
Vps34	Phosphatidylinositol 3-kinase VPS34		
ATG14L	Autophagy-related protein 14L	Member of PI3KC3-C1	
UVRAG	UV radiation resistance associated protein	Member of PI3KC3-C2	
KAP-1	E3 SUMO-protein ligase TRIM28	SUMOylation of Vps34	Yang et al., 2013
DAPK	Death-associated protein kinase	BECN1 phosphorylation	Zalckvar et al., 2009
CUL3	Cullin-3	Poly ubiquitination of ULK1, Vps34, and BECN1	Liu and Chen, 2016
KLHL20	Kelch-like protein 20	Substrate-binding subunit of CUL3 ubiquitin ligase. Recognition of ULK1, Vps34, and BECN1 as substrates	Liu and Chen, 2016
VMP1	Vacuole Membrane Protein 1	Recruitment of PI3KC3-C1 by interaction with BECN1 /autophagosomal membrane	Ropolo et al., 2007
EP300	EP300-interacting inhibitor of differentiation 300	Vps34 acetylation	Su et al., 2017
DFCP1	Double FYVE-containing protein 1	Omegasome marker	Ktistakis and Tooze, 2016
WIPI2b	WD40-repeat phosphoinositide-interacting protein	Isolation membrane marker	Nascimbeni et al., 2017a
ATG12	Autophagy-related protein 12	Member of ATG12-ATG5-ATG16L complex: E3 like function in LC3 conjugation to phosphatidylethanolamine	Klionsky and Schulman, 2014
ATG5	Autophagy-related protein 5		
ATG16L	Autophagy-related protein 16L		
ATG7	Autophagy-related protein 7	E1 in LC3 lipidation and ATG12-ATG5 conjugation	
ATG10	Autophagy-related protein 10	E2 in ATG12-ATG5 conjugation	
ATG3	Autophagy-related protein 3	E2 like function in LC3 lipidation	Satoo et al., 2009
LC3	Microtubule-associated proteins 1A/1B light chain 3B	Vesicle maturation/cargo recognition	Lee and Lee, 2016
SIRT1	NAD-dependent deacetylase sirtuin-1	LC3 deacetylation	Huang et al., 2015
ATG4B	Autophagy-related protein 4B	Clevage of C-terminal Gly of LC3	Maruyama and Noda, 2017
ATG9A	Autophagy-related protein 9 A	Isolation membrane extension	Feng and Klionsky, 2017
Esyt 1, 2, 3	Extended synaptotagmin-1, 2, 3	ER-PM contact sites	Nascimbeni et al., 2017b
AP-4	Adaptor protein 4	Isolation membrane extension	Mattera et al., 2017
Sar1	Sar1	COPII coat: participation in autophagosome biogenesis	Karanasios et al., 2016
Sec 13, 23, 24, 31			
Rab11A	Ras-related protein Rab-11A	Recycling endosomes	Puri et al., 2018
AMBRA1	Activated in BECN1-regulated autophagy protein 1	ULK1 ubiquitination	Nazio et al., 2013
TRAF6	TNF receptor (TNFR)-associated factor 6	ULK1 and BECN1 ubiquitination	Grumati and Dikic, 2018

Moreover, autophagosome biogenesis is regulated by a variety of signaling pathways through posttranslational modification, such as phosphorylations, ubiquitinations, SUMOylations and acetylation, that may account for diverse conditions, functions or selectivity. Furthermore, this molecular regulation, that are eminently druggable, may be relevant in the development of therapeutic strategies of autophagy modulation for complex pathologies such as cancer (Galluzzi et al., 2015) or neurodegenerative diseases (Zare-Shahabadi et al., 2015).

Although there are many aspects still unclear on mammalian autophagosome biogenesis, future findings that shed light on this sophisticated intracellular process can be taken for granted.

## Author Contributions

DG did the literature search, wrote the first draft of the manuscript and designed all the figures. FR wrote a session of the first draft of the manuscript and assisted with the edited version. MV edited and added to the draft of the manuscript and figures and revised the final version of the manuscript.

## Conflict of Interest Statement

The authors declare that the research was conducted in the absence of any commercial or financial relationships that could be construed as a potential conflict of interest.

## Abbreviations

AMBRA1: Activating molecule in BECN1-regulated autophagy protein 1
AMPK: AMP-activated Kinase
AP-4: Adaptor protein 4
ATF6: Activating transcription factor 6
ATG: Autophagy related gen or protein
Bcl-2: B-cell lymphoma 2
BECN1: Coiled-Coil Moesin-Like BCL2-Interacting Protein
BH3: Bcl-2 homology 3
BiP: Binding immunoglobulin protein
BNIP3: BCL2 interacting protein 3
CaMKKB: Calcium/calmodulin-dependent protein kinase kinase 2
COPII: Coat complex protein II
CTAGES5: Cutaneous T-cell linphoma-associated antigen 5
CUL3: Cullin-3
DAPK: Death-associated protein kinase
DEPTOR: DEP domain containing mTOR-interacting protein
DFCP1: Double FYVE containing protein 1
EP300: Histone acetyltransferase p300
ERK1/2: Mitogen-activated protein kinase
Esyt: Extended synaptotagmin
FIP200: FAK family-interacting protein of 200 kDa (also known as RB1CC1)
FOXO3: Forkhead box protein O3
GSK3: Glycogen synthase kinase 3
HIF1α: Hypoxia-inducible factor 1 alpha
HORMA: Hop/Rev7/Mad2 domain
IDR: Intrinsically disordered region
IRE1α: Inositol-requiring enzyme 1 alpha
JNK: c-Jun N-terminal kinases
KAP1: E3 SUMO-protein ligase TRIM28
KHLH20: Kelch-like protein 20
LC3: Microtubule-associated proteins 1A/1B light chain 3B (also known as MAP1LC3B)
LIR: LC3-interacting region
LKB1: Serine/threonine-protein kinase STKB1
MIT: Microtubule interacting and trafficking domain
mLST8: mammalian Letal with SEC13 protein 8
NEDD4: Neural precursor cell expressed developmentally down-regulated protein 4
NEDD4L: Neural precursor cell expressed developmentally downregulated gene 4-like
NRF2: Nuclear factor erythroid 2-related factor 2
PDK1: 3-phosphoinositide-dependent protein kinase 1
PERK: Proline-rich receptor-like protein kinase
PI3K: Phosphatidylinositol 3-kinase
PI3P: Phosphatidylinositol 3-phosphate
PKCδ: Protein kinase C delta type
PRAS40: Proline-rich Akt substrate of 40 kDa
PROPPIN: β-propeller that bind polyphosphoinositides
RAB: Ras-related protein
RAPTOR: Regulatory-associated protein of mTOR
Rheb: Ras homolog enriched in brain
ROS: Reactive oxygen species
SERCA: sarco/endoplasmic reticulum Ca2+-ATPase
SIRT1: Sirtuin 1
SQSTM1: Sequestosome 1 (also known as p62)
STX17: Sintaxin 17
SUMO: Small ubiquitin-like modifier
TFEB: Transcription factor EB
TIP60: 60 KDa Tat-Interactive Protein
mTOR: mammalian Target of Rapamycin
TRAF6: Tumor necrosis factor receptor (TNFR)-associated factor 6
TRAPPIII: transport protein particle (TRAPP) III complex
TSC1/2: Tuberous sclerosis 1/2
ULK1: unc-51-like kinase 1
UVRAG: UV radiation resistance associated protein
VMP1: Vacuole membrane protein 1
Vps: Vacuolar protein sorting
WIPI: WD repeat domain phosphoinositide-interacting protein.
